# Machine learning based radiomics model to predict radiotherapy induced cardiotoxicity in breast cancer

**DOI:** 10.1002/acm2.14614

**Published:** 2024-12-20

**Authors:** Amin Talebi, Ahmad Bitarafan‐Rajabi, Azin Alizadeh‐asl, Parisa Seilani, Benyamin Khajetash, Ghasem Hajianfar, Meysam Tavakoli

**Affiliations:** ^1^ Department of Medical Physics School of Medicine Iran University of Medical Sciences Tehran Iran; ^2^ Cardiovascular Intervention Research Center Rajaie Cardiovascular Medical and Research Center Iran University of Medical Sciences Tehran Iran; ^3^ Echocardiography Research Center, Rajaie Cardiovascular Medical and Research Center Iran University of Medical Sciences Tehran Iran; ^4^ Rajaei Cardiovascular Medical and Research Center Cardio‐Oncology Research Center Iran University of Medical Science Tehran Iran; ^5^ Department of Medical physics Iran University of Medical Sciences Tehran Iran; ^6^ Division of Nuclear Medicine and Molecular Imaging Geneva University Hospital Geneva Switzerland; ^7^ Department of Radiation Oncology, and Winship Cancer Institute Emory University Atlanta Georgia USA

**Keywords:** cardiotoxicity, echocardiography, machine learning, prediction, radiomics, radiotherapy

## Abstract

**Purpose:**

Cardiotoxicity is one of the major concerns in breast cancer treatment, significantly affecting patient outcomes. To improve the likelihood of favorable outcomes for breast cancer survivors, it is essential to carefully balance the potential advantages of treatment methods with the risks of harm to healthy tissues, including the heart. There is currently a lack of comprehensive, data‐driven evidence on effective risk stratification strategies. The aim of this study is to investigate the prediction of cardiotoxicity using machine learning methods combined with radiomics, clinical, and dosimetric features.

**Materials and methods:**

A cohort of 83 left‐sided breast cancer patients without a history of cardiac disease was examined. Two‐ and three‐dimensional echocardiography were performed before and after 6 months of treatment to evaluate cardiotoxicity. Cardiac dose‐volume histograms, demographic data, echocardiographic parameters, and ultrasound imaging radiomics features were collected for all patients. Toxicity modeling was developed with three feature selection methods and five classifiers in four separate groups (Dosimetric, Dosimetric + Demographic, Dosimetric + Demographic + Clinical, and Dosimetric + Demographic + Clinical + Imaging). The prediction performance of the models was validated using five‐fold cross‐validation and evaluated by AUCs.

**Results:**

58% of patients showed cardiotoxicity 6 months after treatment. Mean left ventricular ejection fraction and Global longitudinal strain decreased significantly compared to pre‐treatment (*p*‐value *< *0.001). After feature selection and prediction modeling, the Dosimetric, Dosimetric + Demographic, Dosimetric + Demographic + Clinical, Dosimetric + Demographic + Clinical + Imaging models showed prediction performance (AUC) up to 73%, 75%, 85%, and 97%, respectively.

**Conclusion:**

Incorporating clinical and imaging features along with dose descriptors are beneficial for predicting cardiotoxicity after radiotherapy.

## 1 INTRODUCTION

Breast cancer is one of the leading causes of cancer related death among women worldwide.^1^ The Radiotherapy (RT) is one of the common methods used to treat cancer, improving local disease control and survival.^2^ Although advanced RT leads to improved patient survival, the occurrence of cancer treatment‐induced cardiac dysfunction exhibits considerable variability during treatment. Several studies have examined cardiac complications during RT^3^, ^4^, ^5^ Additionally, in the case of left‐sided breast RT, the placement of the apex and left ventricle (LV) in the treatment field may increase the risk of cardiac side effects such as coronary artery disease, left ventricular dysfunction, myocardial infarction, and arrhythmias, which lead to cardiac mortality.^6^ Therefore, RT‐induced cardiotoxicity is an increasingly important concern in cardio‐oncology.^7^, ^8^, ^9^

In RT, normal tissue complication probability (NTCP) models, such as Lyman–Kutcher–Burman (LKB), relative seriality (RS), and generalized equivalent uniform dose (gEUD), are used to predict the occurrence of toxicity in healthy tissues.^10^ However, these models are limited to dosimetric factors, while patient‐related factors such as demographic, clinical, and image‐derived factors can affect radiation sensitivity and toxicity.^11^ Consequently, considering non‐dosimetric factors can improve prediction accuracy. Moreover, the cost‐effectiveness of a predictive model for anticipating future cardiotoxicity prior to the administration of anti‐cancer treatments is evident.^12^, ^13^

In recent years, machine learning (ML)‐based methods have emerged as contemporary approaches for predicting, identifying, and making decisions without human involvement in different areas, including cardiovascular studies.^14^, ^15^, ^16^ Similarly, radiomics models are powerful tools for characterizing tissue by extracting high dimensional minable data from various medical imaging sources such as magnetic resonance imaging (MRI) and computed tomography (CT).^17^, ^18^, ^19^ Radiomics involves the extraction of numerous quantitative features from images using a high‐throughput approach, transforming them into high‐dimensional data for quantitative analysis.^20^, ^21^ Due to the high number of interdependent features generated, ML algorithms are well‐suited for rendering the best‐performing models. These algorithms can improve decision‐making by considering many parameters.^22^ Several studies have demonstrated the effectiveness of radiomics and ML methods in predicting radiation‐induced toxicity in different cancers, such as head and neck,^23^, ^24^, ^25^ lung,^26^, ^27^ and prostate^28^, ^29^ cancers.

To the best of our knowledge, no ultrasound‐based radiomics research has been conducted on predicting RT‐induced cardiotoxicity. This prospective study aimed to establish a ML‐based radiomics model using echocardiographic images along with demographic, dosimetric, and clinical features to predict cardiotoxicity in left‐sided breast cancer patients after RT. Our contributions in this study are: (1) Investigating the capability of ML classifiers in combination of various feature selection algorithms to predict cardiotoxicity in breast cancer patients. (2) Assessing the combination with dosimetric, clinical, and ultrasound radiomics features in improving the accuracy of ML model predictions.

## 2 MATERIALS AND METHODS

**FIGURE 1:**
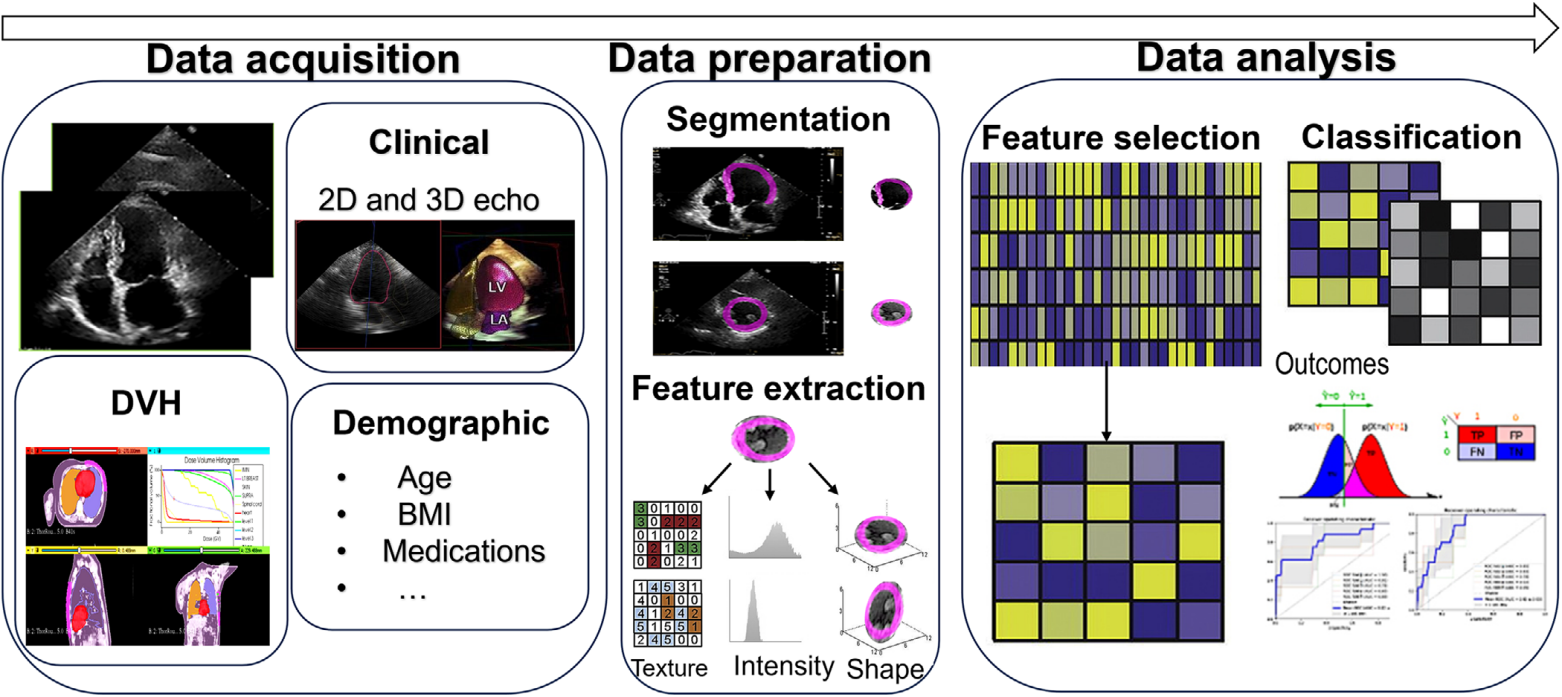
Schematic design of the study workflow.

The entire workflow of the current study is presented in Figure 1 and explained in the following subsections.

### 2.1 Patient selection

In a prospective cohort study, 83 left‐sided breast cancer patients, separated into two groups– without and with toxicity– were included from November 2018 to June 2021. Exclusion criteria included a history of heart disease, being in the metastatic phase of cancer, renal diseases, congestive heart failure symptoms, a history of chemotherapy, severe or uncontrolled arterial hypertension, and abnormalities in the electrocardiogram (ECG) (e.g., abnormal rhythm, bundle branch blocks). After obtaining informed consent, demographic information was recorded prior to the start of the study.

### 2.2 Treatment

Before radiotherapy, a CT scan was taken of each patient for treatment planning. Patients were treated using a three‐dimensional conformal radiation therapy (3D‐CRT) technique. The prescribed dose for all patients ranged between 45 and 50 Gy, delivered in 25 fractions at 2 Gy per fraction. In the case of conservative breast surgery, an additional boost dose of 10 Gy in 5 fractions was delivered to the target.

### 2.3 Imaging

Two‐ and three‐dimensional transthoracic ultrasound echocardiography in conjunction with speckle tracking mode were performed for each patient before and 6 months after completion of treatment. An echocardiographic specialist performed imaging according to the guidelines of the American Society of Echocardiography using an EPIQ‐7 Philips ultrasound machine. Left ventricle ejection fraction (LVEF) was assessed using the heart model tool, which provides robust and reproducible ejection fraction measurements. In addition, functional diastolic and systolic parameters were recorded at each imaging stage. According to the guidelines, a 10% reduction in LVEF to less than 55% in the post‐treatment echo compared to the baseline echo without symptoms, or a 5% reduction in LVEF to less than 50% with symptoms, was considered cardiotoxicity.^30^

### 2.4 Feature extraction

Pre‐treatment echocardiographic data used for clinical feature extraction were collected in DICOM format. The LV was manually segmented by a specialist, with short‐axis views (SAX) at the end‐systole (ES) and end‐diastole (ED) frames according to ECG. Statistical texture features from each frame were extracted using LIFEx software.^31^

Details of all the features presented in Table 1. In the same direction, ultrasound echocardiography imaging features from different sets, including gray level co‐occurrence matrix (GLCM), gray level run length matrix (GLRLM), neighborhood grey‐level difference matrix (NGLDM), grey‐level zone length matrix (GLZLM), and conventional features (Min, Max, Mean), were extracted from echocardiographic images. Collectively, 278 imaging features were extracted from each patient.

**TABLE 1:** Comparison of functional echocardiographic parameters in the toxicity group before and after 6 months of treatment (* Significant).

Parameter	Pre‐treatment (Mean—SD.)	Post‐treatment (Mean—SD.)	*p*‐value
LV‐EDD (mm)	44.30–4.51	45.06–2.60	0.43
IVS (mm)	7.73–1.15	8.10–0.90	0.18
RV‐EDD (mm)	28.32–3.13	28.12–2.75	0.72
E‐Velocity (cm/s)	81.35–14.40	69.80–13.82	0.001*
A‐Velocity (cm/s)	74.57–17.83	69.29–11.94	0.18
RVS‐Velocity (cm/s)	11.79–1.98	11.61–1.40	0.63
TAPSE (mm)	21.72–2.53	21.61–2.80	0.86
LA‐Area (cm2)	16.20–2.71	15.97–2.71	0.59
TR‐Gradient (mmHg)	21.75–3.97	18.35–5.24	0.004*
sPAP (mmHg)	27.80–6.22	23.08–5.19	0.001*
Volume 3D (cm3)	90.18–16.78	91.41–7.29	0.68
LV‐EF by Heart Model	58.92–6.84	49.25–4.45	0.00001*
LV‐ESV	44.18–20.56	45.85–7.91	0.68
LV‐EDV (cm3)	89.08–15.81	91.20–14.68	0.55
S‐Septal (cm/s)	6.74–1.13	7.08–1.27	0.19
E‐Septal (cm/s)	8.65–2.28	8.46–2.07	0.66
E‐lateral (cm/s)	11.85–2.89	11.08–2.48	0.12
LV‐GLS	−19.30–2.97	−17.01–1.52	0.0001*
LV‐ESD (mm)	29.15–5.32	28.27–7.15	0.53
LV‐PWD (mm)	7.74–1.25	8.15–1.25	0.17

Abbreviations: A‐Velocity, peak velocity flow in late diastole, Elateral, Lateral Mitral Annular Velocity, E‐Septal, Septal mitral annulus velocity, E‐Velocity, Early ventricular filling Velocity, IVS, Interventricular Septum, LA‐Area, Left Atrium Area, LV‐EDD, Left Ventricle End Diastole Diameter, LV‐EDV, Left Ventricle End Diastole Volume, LVEF by Heart Model, Left Ventricle Ejection Fraction measured in Heart Model, LV‐ESD, Left Ventricular End‐Systolic Diameter, LV‐ESV, Left ventricle End Systole Volume, LV‐GLS, Left ventricle Global Longitudinal Strain, LV‐PWD, Left ventricular posterior wall thickness, RV‐EDD, Right Ventricle End Diastole Diameter, RVS‐Velocity, Right Ventricle Systolic Excursion Velocity, sPAP, systolic pulmonary pressure, S‐Septal, Peak velocity of systolic pulmonary vein flow, TAPSE, Tricuspid annular plane systolic excursion, TR‐Gradient, tricuspid regurgitation, Volume 3D, Left ventricle volume (measured in 3D mode).

For all patients, the age distribution ranged from 26 to 78 years, with a mean age of 49. Patients’ demographics included Body Mass Index (BMI) *> *25, family history of cancer, breast surgery, smoking status, history of diabetes, history of hypertension, and use of aldactone, atorvastatin, and lisinopril. A total of 10 Demographic features extracted for each patient. The details of all features are summarized in the Table 2.

**TABLE 2:** The details of demographic features respect to number of patients.

Age	26‐36 (12)	37‐46 (24)	47‐56 (21)	57‐66 (16)	67‐78 (10)
**BMI ** *>* 25	7	16	14	11	7
**Family history cancer**	7	12	10	10	4
**Breast surgery**	3	10	5	8	1
**Smoking status**	0	1	1	0	0
**Diabetes history**	0	1	1	3	0
**Hypertension history**	0	1	2	5	2
**Taking aldactone**	2	2	1	0	0
**Taking atorvastatin**	1	4	2	1	1
**Taking lisinopril**	5	2	1	4	2

Abbreviation: BMI, body mass index.

**FIGURE 2:**
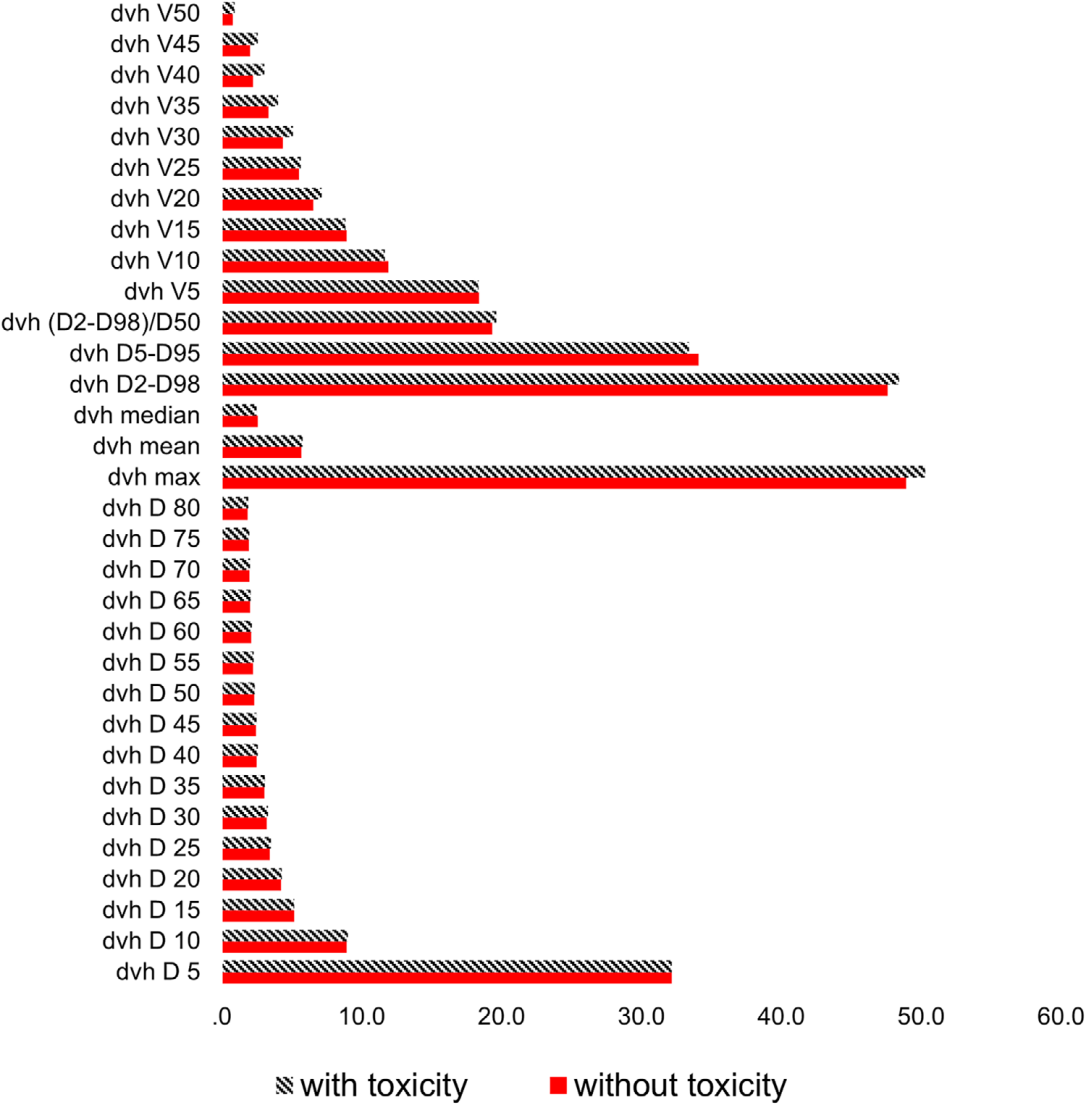
Comparison of dosimetric features extracted from the DVH for patients with and without toxicity. DVH, dose‐volume histogram.

Dosimetric calculations were performed for the whole heart. Quantitative data were extracted from dose‐volume histogram (DVH), encompassing cardiac device volumes (cc) receiving doses ranging from 0.01 Gy (*V*_0_ *_._*_01_ *_Gy_*) to 50 Gy (*V*_50_ *_Gy_*). Additional treatment planning parameters obtained for each patient included heart volume (cc), maximum, minimum, modal, median, and mean doses (Gy), as well as equivalent diameter (cm). Overall, 32 DVH‐related characteristics were derived for each patient. The Figure 2 represents these features in each group of patients (with and without toxicity)

### 2.5 Feature selection and predicting models

Given the large number of extracted features, it was crucial to perform feature selection prior to model computation to remove redundant and irrelevant features, thereby reducing the risk of overfitting. In this study, we applied three different feature selection algorithms: forward selection (FS), maximum relevance minimum redundancy (MRmr), and recursive feature elimination (RFE).

During the feature selection process, features were ranked from 0 to 1 based on their relevance to the prediction task. Instead of predefining a specific number of features to select, we adopted an importance‐based approach. Features were selected if their average normalized importance score was ≥ 0.9, where the importance was defined differently according to the selection technique used. For FS and RFE, importance scores were derived from model performance metrics (AUC) and SVM coefficients, respectively. For MRmr, importance was calculated using mutual information scores. This process was performed within each fold of a five‐fold cross‐validation framework. After feature selection in each fold, we aggregated the results across all folds to identify the final set of features. Only features with an average importance score of ≥ 0.9 across all folds were retained. By using a high feature importance threshold, we prioritized only the most predictive features, which improves model interpretability and ensures that the strongest predictors of cardiotoxicity are included. This is especially critical in healthcare‐related models, where understanding the contributing factors is as important as achieving high prediction accuracy. Moreover, setting a higher threshold helps to minimize the risk of overfitting, particularly given the relatively small size of the dataset, by reducing the likelihood of selecting less significant features.

Prior to the model selection step, we divided the dataset into a training and validation set (comprising 70% of the data) and reserved the remaining 30% for testing. Prediction modeling was performed using five different classifiers: neural network (NN), generalized linear model (GLM), Naive Bayes (NB), support vector machine (SVM), and random forrest (RF). An internal five‐fold cross‐validation method was used to validate the models and obtain the area under the ROC curve (AUC). We compared the performance of prediction of radiation‐induced cardiotoxicity in across four different groups:

Dosimetric, where only dose‐volume parameters from DVH were considered in the modeling.
Dosimetric + demographics, where demographic properties such as age, BMI, and others were included in the models.
Dosimetric + demographics + clinical, where echocardiographic parameters (see, Table 1) were included.
Dosimetric + demographics + clinical + imaging, where imaging radiomic were included to evaluate their impact on prediction performance.

## 3 RESULTS

### 3.1 Echocardiographic measurements

After 6 months of RT, 58.5% of patients developed a reduction in LVEF considered cardiotoxicity according to the guidelines. LVEF was assessed using a three‐dimensional heart mode, which allows for more precise contouring of the actual shape of the LV cavity and does not rely on the geometric assumptions that two‐dimensional LVEF assessment does. As shown in Table 1, compared to pre‐treatment, there was a significant decrease in early ventricular filling velocity (E‐Velocity) (*p*‐value = 0.001), tricuspid regurgitation gradient (TR‐gradient) (*p*‐value = 0.004), systolic pulmonary pressure (sPAP) (*p*‐value = 0.001), LVEF (*p*‐value *< *0.001), and left ventricle global longitudinal strain (LV‐GLS) (*p*‐value *< *0.001) in post‐treatment echocardiographs. Although left ventricular end diastole diameter (LV‐EDD), interventricular septum (IVS), left ventricle volume (by 3D), left ventricle end systole volume (LV‐ESV), S‐Septal, E‐Septal, and left ventricle posterior wall thickness (LV‐PWD) were increased, the differences were not statistically significant. Additionally, no significant changes were observed in left ventricular end systole diameter (LV‐ESD), left atrium area (LA‐Area), E‐lateral, tricuspid annular plane systolic excursion (TAPSE), RVS—Velocity, and right ventricular end diastole diameter (RV‐EDD).

### 3.2 Selected features

From each group, top‐ranked common features were selected, which means these features had the strongest correlation with the defined label. Results showed that among the selected features across all feature selection algorithms, there were three dosimetric, three clinical, and nine radiomic features that were common between all feature selection techniques. The details of all parameters are presented in Table 3.

**TABLE 3:** Selected features in feature selection techniques.

Feature set	RFE feature	MRmr feature	FS feature
	*V*5*Gy*	*V*30*Gy*	*V*25*Gy*
	*V*10*Gy*	*V*35*Gy*	*V*30*Gy*
**Dosimetric**	*V*30*Gy*	*V*40*Gy*	*V*35*Gy*
	*V*35*Gy*	*V*45*Gy*	*V*40*Gy*
	*V*40*Gy*	*V*50*Gy*	*V*50*Gy*
		*D*20*Gy*	*D*65*Gy*
	LVEDD	LVEDD	E Velocity
	sPAP	IVS	TAPSE
**Clinical**	RVEDD	E Velocity	TR Gradient
	E Velocity	TR Gradient	Volume 3D
	TR Gradient	A Velocity	LVEF by Heart model
	S septal	sPAP	sPAP
	ES‐conventional‐Skewness	ES‐GLRLM‐LRLGE	ES‐GLCM‐Contrast
	ED‐GLRLM‐LRLGE	ES‐GLZLM‐LZLGE	ED‐GLRLM‐SRLGE
	ES‐GLCM‐Contrast	ED‐GLRLM‐SRLGE	ES‐GLRLM‐LRLGE
	ED‐GLRLM‐SRLGE	ED‐GLRLM‐LRLGE	ES‐GLZLM‐LZLGE
	SAX‐ES‐conventional‐std	ES‐conventional‐Skewness	SAX‐ES‐GLZLM‐LZLGE
	ES‐GLRLM‐LRLGE	ES‐discretized‐Skewness	SAX‐ES‐conventional‐std
	SAX‐ES‐GLZLM‐LZLGE	SAX‐ES‐conventional‐Kurtosis	SAX‐ES‐conventional‐Kurtosis
**Radiomics**	SAX‐ED‐GLZLM‐LGZE	ED‐GLZLM‐LGZE	SAX‐ES‐GLRLM‐LGRE
	ES‐GLZLM‐LZLGE	ES‐GLCM‐Contrast	ES‐discretized‐ HISTO‐Entropy
	ED‐conventional‐std	ED‐discretized‐Skewness	ES‐conventional
	SAX‐ES‐GLRLM‐LGRE	SAX‐ES‐GLRLM‐LGRE	ED‐conventional‐Skewness
	SAX‐ED‐GLZLM‐SZLGE	SAX‐ED‐GLZLM‐SZLGE	SAX‐ED‐GLZLM‐LGZE
	SAX‐ES‐GLRLM‐LRLGE	ED‐conventional‐std	ED‐GLRLM‐LRLGE
	SAX‐ES conventional‐Kurtosis	SAX‐ES‐GLZLM‐LZLGE	ES‐conventional‐Skewness

Abbreviations: *D*_20_, *D*_65_, the dose received by 20% and 65% of the heart respectively; E Velocity, Early ventricular filling Velocity; ED, End Diastole; ES, End Systole; GLCM, Gray‐Level Co‐occurrence Matrix; GLRLM, Gray Level Run Length Matrix; GLZLM, Grey‐Level Zone Length Matrix; IVS, Interventricular Septum; LGRE, Low Gray Level Run Emphasis; LRLGE, Long‐Run Low Gray‐Level Emphasis; LVEDD, Left Ventricle End Diastole Diameter; LV‐EF by Heart Model, Left Ventricle Ejection Fraction measured in Heart Model; LZLGE, Long‐ Zone Low Gray Level Emphasis; RVEDD, Right Ventricle End Diastole Diameter; SAX, Short Axis; sPAP, systolic pulmonary pressure; SRLGE, Short‐Run Low Gray Level Emphasis; TR Gradient, tricuspid regurgitation, *V*_5_ *_Gy_*, *V*_10_ *_Gy_*, *V*_25_ *_Gy_*, *V*_30_ *_Gy_*, *V*_35_ *_Gy_*, *V*_40_ *_Gy_*, *V*_45_ *_Gy_*, *V*_50_ *_Gy_*,, the percentage of cardiac volume receiving 30, 35 and 40 Gy respectively; Volume 3D, Left ventricle volume (measured in 3D mode).

### 3.3 Cardiotoxicity prediction

The findings in this study are reported under four major feature groups: (1) dosimetric, (2) combination of dosimetric and demographic, (3) dosimetric, demographic, and clinical, and (4) all features including dosimetric, demographic, clinical, and imaging.

#### 3.3.1 Group1: Dosimetric

In the first group, cardiotoxicity was predicted using dosimetric data. According to whole heart dose distribution, mean heart dose was 5.31–0.98 Gy, and maximum heart dose ranged from 48.2 Gy to 53.2 Gy. A comparison of dosimetric parameters between the two groups of patients showed that *V*_30_ *_Gy_*, *V*_35_ *_Gy_*, and *V*_40_ *_Gy_* were significantly different. Table 4 depicts the average AUCs between all folds for the cross‐combination of feature selectors and classifiers. As shown, the best results in this group were observed with RFE feature selection and the classifiers SVM and RF with AUCs of 0.72 and 0.73, respectively. Moreover, in Table 5, we have presented the AUC results for the RF classifier, split across each of the five folds.

**TABLE 4:** The cross‐combination of different feature selection methods and classifiers in predicting cardiotoxicity using dosimetric data.

Classifier	GLM	NB	RF	SVM	NN
**FS**	0.68	0.65	0.63	0.68	0.68
**MRmr**	0.58	0.57	0.57	0.60	0.57
**RFE**	0.63	0.62	**0.73**	0.72	0.57

Abbreviations: FS, forward Selection; GLM, generalized linear model; MRmr, maximum relevance minimum redundancy; NB, Na¨ıve Bayes; NN, neural network; RFE, recursive feature elimination; SVM, support vector machine.

**TABLE 5:** The cross‐combination of various feature selection methods and the RF classifier in Table 4 yielded the best results in all folds.

Classifier	Feature selection	Fold 1	Fold 2	Fold 3	Fold 4	Fold 5	Average
**RF**	**FS**	0.63	0.69	0.63	0.62	0.58	0.63
	**MRmr**	0.53	0.61	0.53	0.54	0.55	0.57
	**RFE**	0.76	0.72	0.70	0.67	0.76	**0.73**

Abbreviations: FS, forward Selection; MRmr, maximum relevance minimum redundancy; RFE, recursive feature elimination.

#### 3.3.2 Group2: Dosimetric + Demographic

The model's performance (the average AUCs between all folds) from the cross‐combination of feature selection and classification approaches for the demographic and dosimetric data groups is depicted in Table 6. The results show that the prediction performances range from 0.60 to 0.75, with the combinations of SVM + MRmr and RF + FS have the highest performance (AUC = 0.75).

**TABLE 6:** The predictive performance (AUC) of feature selection and classification methods of dosimetric and demographic data group.

	GLM	NB	RF	SVM	NN
**FS**	0.72	0.69	**0.75**	0.72	0.72
**MRmr**	0.66	0.65	0.67	**0.75**	0.60
**RFE**	0.69	0.69	0.65	0.73	0.66

Abbreviations: FS, forward Selection; GLM, generalized linear model; MRmr, maximum relevance minimum redundancy; NB, Na¨ıve Bayes; NN, neural network; RFE, recursive feature elimination; SVM, support vector machine.

Moreover, in Table 7, we have presented the AUC results for the SVM classifier, split across each of the five folds.

**TABLE 7:** The cross‐combination of various feature selection methods and the SVM classifier in Table 6 yielded the best results in all folds.

Classifier	Feature selection	Fold 1	Fold 2	Fold 3	Fold 4	Fold 5	Average
**SVM**	**FS**	0.80	0.66	0.68	0.67	0.77	0.72
	**MRmr**	0.82	0.65	0.67	0.80	0.80	**0.75**
	**RFE**	0.77	0.65	0.75	0.67	0.80	0.73

Abbreviations: FS, forward Selection; MRmr, maximum relevance minimum redundancy; RFE, recursive feature elimination; SVM, support vector machine

#### 3.3.3 Group3: Dosimetric + Demographic + Clinical

The cross‐combination models for predicting cardiotoxicity using demographic and clinical data with dosimetric parameters are shown in Table 8 for the average AUCs of all folds. As indicated, including clinical data (Table 1), the model improved the prediction performance. The best performance in this group was observed with RFE feature selection with the combination of SVM, RF, and NN classifiers with AUCs of 0.85, 0.84, and 0.84, respectively. Furthermore, in Table 9, we have presented the AUC results for the best model, the SVM classifier, split across each of the five folds.

**TABLE 8:** The predictive performance (AUC) of feature selection and classification methods of dosimetric, demographic, and clinical data group.

	GLM	NB	RF	SVM	NN
**FS**	0.75	0.68	0.76	0.66	0.75
**MRmr**	0.62	0.50	0.76	0.63	0.50
**RFE**	0.81	0.79	0.84	**0.85**	0.84

Abbreviations: FS, forward Selection; GLM, generalized linear model; MRmr, maximum relevance minimum redundancy; NB, Na¨ıve Bayes; NN, neural network; RFE, recursive feature elimination; SVM, support vector machine.

**TABLE 9:** The cross‐combination of various feature selection methods and the SVM classifier in Table 8 yielded the best results in all folds.

Classifier	Feature selection	Fold 1	Fold 2	Fold 3	Fold 4	Fold 5	Average
**SVM**	**FS**	0.60	0.60	0.77	0.58	0.77	0.66
	**MRmr**	0.57	0.58	0.66	0.60	0.74	0.63
	**RFE**	0.83	0.82	0.88	0.81	0.91	**0.85**

Abbreviations: FS, forward Selection; MRmr, maximum relevance minimum redundancy; RFE, recursive feature elimination; SVM, support vector machine.

#### 3.3.4 Group4: Dosimetric + Demographic + Clinical + Imaging

In the final group, cardiotoxicity was modeled using imaging radiomic features along with Dosimetric, demographic and clinical data. The average AUCs results of all folds for this group are shown in Table 10. On average, prediction performance in this group was exceptional. The best result was observed with the combination of RFE feature selection and the SVM classification method with an AUC of 0.97.

**TABLE 10:** The predictive performance (AUC) of feature selection and classification methods of dosimetric, demographic, clinical, and imaging data group.

	GLM	NB	RF	SVM	NN
**FS**	0.80	0.82	0.77	0.82	0.82
**MRmr**	0.80	0.67	0.96	0.93	0.67
**RFE**	0.95	0.76	0.90	**0.97**	0.90

Abbreviations: FS, forward Selection; GLM, generalized linear model; MRmr, maximum relevance minimum redundancy; NB, Na¨ıve Bayes; NN, neural network; RFE, recursive feature elimination; SVM, support vector machine.

In Table 11, we have presented the AUC results for the best model, SVM classifier, split across each of the five folds.

**TABLE 11:** The cross‐combination of various feature selection methods and the SVM classifier in Table 10 yielded the best results in all folds.

Classifier	Feature selection	Fold 1	Fold 2	Fold 3	Fold 4	Fold 5	Average
**SVM**	**FS**	0.82	0.80	0.83	0.80	0.83	0.82
	**MRmr**	0.82	0.96	0.96	0.86	0.91	0.93
	**RFE**	0.95	0.98	0.97	0.94	0.99	**0.97**

Abbreviations: FS, forward Selection; MRmr, maximum relevance minimum redundancy; RFE, recursive feature elimination; SVM, support vector machine.

**FIGURE 3:**
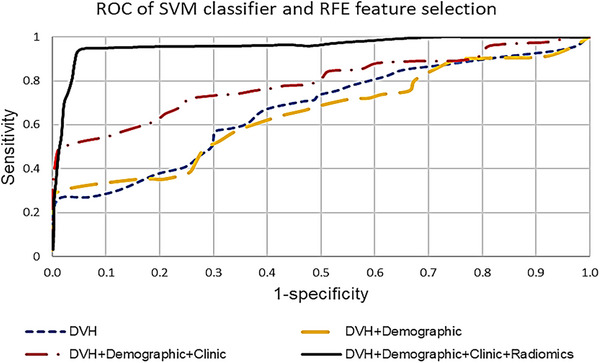
Comparison ROC plots between different feature groups for RFE feature selection and SVM classifier. RFE, recursive feature elimination; SVM, support vector machine.

Following above results, Rthe ROC curves for different model groups are compared in Figure 3 specifically for the RFE feature selection and SVM classifier.

**FIGURE 4:**
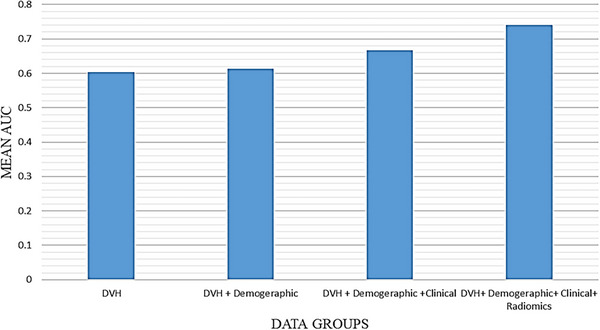
Plot comparing the predictive performance (mean AUC) of all groups.

Moreover, by averaging all AUCs from the different groups, Figure 4 presents an average comparison of different feature groups. As has been shown, adding new data to models improves the prediction performance of cardiotoxicity. This improvement is particularly evident when radiomics data included in the modeling process.

## 4 DISCUSSION

In this study, we investigated the potential to improve the prediction of radiation‐induced cardiotoxicity after RT in left‐sided breast cancer patients. Radiation therapy is a significant source of cardiac complications in breast cancer treatment. The risk of radiation‐induced cardiotoxicity increases when a portion of the left ventricle is within the radiation field. Studies have shown that high absorbed doses to the heart are associated with severe cardiotoxicity. Our results indicated that cardiac dose is higher in patients who experienced cardiotoxicity.^32^

In the context of prediction outcomes, radiobiological models are used clinically to predict complications. These models estimate the probability of normal tissue complications based on dose and irradiated volume. However, their accuracy is limited because they do not adequately consider effective risk factors such as patient characteristics and clinical information. Previous studies have shown that most normal tissue complications are attributed to patient‐related factors.^33^ Early detection of RT‐induced cardiotoxicity and ultimately the prevention of clinical congestive heart failure remains ongoing challenges in clinical oncology. Echocardiography is the preferred imaging technique for monitoring and diagnosing cardiotoxicity related cancer treatment. It is widely available and can assess systolic and diastolic function.^34^ Currently, cardiac imaging biomarkers for detecting myocardial dysfunction during and after cancer treatment are LV‐EF and LV‐GLS.^35^ According to the American Society of Echocardiography and the European Association of Cardiovascular imaging a 10% reduction in LVEF to a LVEF *< *55%, constitutes cardiotoxicity.^30^ The present study showed that these parameters decrease in patients after treatment.

Conversely, advances in medical image analysis have enabled the high‐throughput extraction of quantitative features. Radiomics involves converting digital images into high‐dimensional data and ensuring data mining to support clinical decision‐making. In recent years, many studies have been done for the assessment of RT induced normal tissue complications in head and neck,^19^, ^36^ thoracic,^37^ and prostate^29^ RT. The present study's findings showed that incorporating radiomics features and clinical parameters can improve the prediction performance of ML‐based models. The results show that the predictive value can improve when using demographic, clinical, and radiomic features in the model (Figure 4). When building a ML model, adding redundant variables reduces the generalization capability of the model and may also reduce the overall accuracy of a classifier. Also, using different feature selection and classification methods helps for successful realization of radiomics‐based predictive analyses. The FS method incrementally adds features to the model based on their ability to improve the chosen performance metric (AUC). The RFE method starts with all features and eliminates them one by one, based on their impact on model performance. It usually works well with linear models like SVM. MRmr selects features that have high mutual information with the target variable and low redundancy with other features. It often captures non‐linear relationships but may not always align well with all classifiers.

In Group 1, SVM generally performed better with the FS and RFE feature sets, achieving AUC values of 0.68 and 0.72, respectively. This suggests that SVM benefits from linearly separable features but may struggle with features selected by MRmr, which capture more complex, non‐linear patterns. Both FS and RFE tend to identify features that contribute to clear decision boundaries, which is ideal for SVM. Our findings indicate that the *V*_30_ *_Gy_*, *V*_35_ *_Gy_*, and *V*_40_ *_Gy_* factors have strong discriminative power in predicting cardiotoxicity. These dosimetric parameters represent the volume of the heart receiving at least 30 Gy, 35 Gy, and 40 Gy of radiation, respectively—commonly used dose thresholds to evaluate the safety of heart exposure during RT. Higher values of *V*_30_ *_Gy_*, *V*_35_ *_Gy_*, and *V*_40_ *_Gy_* indicate a larger heart volume receiving these doses, which increases the risk of long‐term heart complications.^32^ The consistent presence of *V*_30_ *_Gy_*, *V*_35_ *_Gy_*, and *V*_40_ *_Gy_* across all techniques suggests that these features are crucial for cardiotoxicity prediction. The RF performed best with the RFE feature set (AUC 0.73), likely because RFE selected features that help reduce overfitting and improve generalization. Conversely, the NB performed poorly across all feature sets. Since NB assumes feature independence, the dependencies between selected features likely caused its lower performance.

In Group 2, demographic parameters were added to the analysis. They could have influenced the interactions among the dosimetric features. The ML‐based models can implicitly benefit from such feature interactions, improving predictive power. The inclusion of demographic data might have altered the relationships between dosimetric features and the target variable (cardiotoxicity), leading to more informative feature combinations, even if the demographic features themselves were not selected. Several studies have explored the predictive power of echocardiographic parameters, such as strain and EF, for LV^38^, ^39^ and RV cardiotoxicity.^40^ Clinical echocardiographic parameters are direct indicators of heart function and structural integrity, and they are likely to correlate more strongly with cardiotoxicity than dosimetric or demographic data alone. Parameters like E‐Velocity, sPAP, TAPSE, and LVEF are key markers of heart function. Their inclusion provides the model with specific information on cardiac health, aiding more accurate cardiotoxicity prediction.

In Group 3, RF and SVM models achieved AUCs of up to 0.84 and 0.85, respectively, when using the RFE feature set. This suggests that the features selected by RFE were highly effective for both models, which are adept at handling complex feature interactions. The inclusion of echocardiographic features likely introduced relevant non‐linear interactions that RF and SVM could leverage better than simpler models like GLM or NB. GLM combined with FS achieved an AUC of 0.75. While GLM is a linear model, FS likely selected features with strong individual linear relationships to the target variable (cardiotoxicity). NB's performance remained relatively low across all feature selection techniques, reflecting its limitation in modeling datasets where feature independence assumptions are violated, as often happens with correlated echocardiographic parameters. AUC values for NB ranged from 0.50 to 0.79.

Our results indicated that certain pre‐treatment echocardiographic parameters, such as E‐Velocity, TR Gradient, and sPAP, have the potential to predict cardiotoxicity in breast cancer patients. Tuohinen et al.^41^ found that radiation‐associated changes in diastolic parameters could be observed after three years of treatment. Several studies have also reported a decrease in E‐Velocity after RT,^42^, ^43^ signaling diastolic dysfunction, which reflects impaired heart relaxation and filling. However, echocardiographic parameters related to diastolic function depend on multiple factors, including cardiac preload, rhythm, age, and measurement techniques.^44^ Additionally, sPAP is linked to pulmonary artery pressure during right ventricular contraction and is an indicator of pulmonary hypertension. In our study, we observed that higher pre‐treatment sPAP levels were associated with a higher risk of cardiotoxicity. TR Gradient measures the pressure difference between the right ventricle and the right atrium, and it may serve as a predictor of cardiotoxicity, particularly in relation to right heart dysfunction.

The inclusion of radiomics features in the final stage of analysis significantly improved model performance, especially for complex classifiers like RF and SVM. Radiomics features, particularly those related to texture (e.g., GLCM and GLRLM), capture detailed information about tissue heterogeneity and structural changes that may not be visible using traditional clinical or dosimetric parameters. Cardiovascular changes due to RT, such as myocardial fibrosis, may manifest as textural changes in cardiac imaging, which radiomics features can detect. These features offer a more granular understanding of cardiotoxicity risk. Models like GLM, NB, SVM, and NN all performed equally well with FS, achieving AUC values ranging from 0.80 to 0.82. FS selected a concise set of radiomics features, such as GLCM‐Contrast and GLRLM‐SRLGE, which are strong individual predictors of cardiotoxicity. The relatively high linear relationships of these features with the outcome explain why simpler models like GLM and NB performed well. However, RF performance was lower with FS (AUC 0.77), possibly because FS may not capture the complex feature interactions that RF is more capable of leveraging.

RFE combined with SVM yielded the best performance (AUC 0.97), followed by GLM (AUC 0.95) and RF (AUC 0.90). This suggests that RFE identified an optimal feature set that captured a broader range of features, including ES GLRLM‐LRLGE, ED‐GLZLMLGZE, and SAX‐ES‐GLRLM‐LGRE, which likely drove these improvements. RFE is effective because it iteratively eliminates less informative features, leaving only the most relevant, particularly for complex, non‐linear models like RF and SVM. NN also performed well with RFE (AUC 0.90), likely benefiting from the rich texture and structural information encoded in the radiomics features. MRmr combined with RF and SVM achieved AUCs of 0.96 and 0.93, respectively, demonstrating MRmr's ability to select features with high relevance while minimizing redundancy. This led to improved performance in models capable of modeling complex feature interactions. However, NB and NN performed poorly with MRmr (AUC 0.67), likely because the selected features had more complex relationships that these models could not leverage as effectively.

In the context of predicting cardiotoxicity, our results showed that nine radiomics features extracted from different cardiac views (SAX view) and cardiac phases (ES and ED) demonstrated potential in predicting cardiotoxicity and were selected in all feature selection techniques. Seven of these radiomics features were from the ES phase, suggesting that this phase may be particularly important for detecting cardiac structural changes related to cardiotoxicity. During the ES phase, the heart is in its most contracted shape, which can make structural abnormalities such as fibrosis or scar tissue more apparent. On the other hand, ED features could help identify patients at risk of diastolic dysfunction, a common side effect of cardiotoxicity. Four of the selected radiomics features are from the GLRLM, indicating the significance of these features in predicting cardiotoxicity. GLRLM features provide information about the texture of cardiac tissue by analyzing the lengths of intensity runs. LRLGE and SRLGE focus on the distribution of long and short runs with low‐intensity values, respectively. Additionally, the inclusion of GLZLM features, which indicate areas of contiguous pixels with the same gray level, allows for the identification of larger homogeneous regions within the cardiac muscle. This can be indicative of tissue uniformity or abnormalities such as fibrosis. Skewness and kurtosis features provide information about the distribution of pixel intensities in cardiac imaging. The selection of these features highlights the potential of statistical properties of texture, such as asymmetry and peakedness, in predicting cardiotoxicity.

On the other hand, features like ED‐GLRLM‐SRLGE and SAX‐ES‐GLZLM‐LZLGE are selected for their relevance to individual pixel‐level texture, capturing highly specific radiomics data patterns. FS emphasizes features that individually contribute the most to the model, which explains the moderate performance improvements seen in simpler classifiers like NB and GLM. The selection of unique features like SAX‐ES‐CONVENTIONAL‐Kurtosis through RFE enhances the performance of complex models such as SVM and RF, which rely on the interactions and combinations of multiple feature types (e.g., shape, texture). Additionally, some of the unique features chosen by MRmr, such as SAX‐ED‐GLZLM‐SZLGE and SAX ES CONVENTIONAL‐Std, focus on minimizing feature redundancy, thereby increasing the robustness of the RF and SVM models.

Finally, this research demonstrated how incorporating patient‐based characteristics, quantitative features extracted from images, dosimetric parameters, and ML methods can enhance the accuracy of predicting cardiotoxicity in breast cancer patients. Although the results were promising, further studies with larger sample sizes are needed to validate these findings. Additionally, this investigation primarily focused on LV cardiotoxicity, but cardiotoxicity is not limited to this area. Future studies could be expanded to include other structures related to cardiotoxicity, such as the RV and cardiac valves, in radiomic analysis to provide a more comprehensive understanding of cardiotoxicity induced by RT. Furthermore, while transthoracic echocardiography (TTE) is recommended by various guidelines for patients at increased cardiovascular risk, cardiac magnetic resonance (CMR) imaging, with its superior image quality and capability to reduce segmentation errors, could be valuable for assessing the predictive performance of radiomic features in cardiotoxicity. In conclusion, combining radiomics with ML algorithms can yield relevant data to improve predictive models for radiation‐induced toxicities.

## AUTHOR CONTRIBUTIONS

This research originated from the combined ideas of Amin Talebi, Ahmad Bitarafan‐Rajabi, and Meysam Tavakoli. Data collection involved a team of researchers (Amin Talebi, Ahmad Bitarafan‐Rajabi, Azin Alizadeh‐asl, Parisa Seilani, Benyamin Khajetash, Ghasem Hajianfar). Amin Talebi conducted the statistical analysis and interpretation of data, with quality control ensured collaboratively with Meysam Tavakol. Manuscript preparation and revision were undertaken through collaboration among all authors, with Meysam Tavakoli overseeing the entire project.

## CONFLICT OF INTEREST STATEMENT

The authors have no relevant conflict of interest to disclose.

## ETHICS STATEMENT

All ethical issues relating to the patients are approved by the ethical committee of Iran University of Medical Sciences. Written informed consent was obtained from all participants. All procedures performed in studies involving human participants were in accordance with the 1964 Helsinki Declaration and its later amendments.

## Data Availability

The data used and/or analyzed during the current study are available from the corresponding author on reasonable request.
